# Alcohol Consumption, Hangovers, and Smoking among Buenos Aires University Students during the COVID-19 Pandemic

**DOI:** 10.3390/jcm12041491

**Published:** 2023-02-13

**Authors:** Analia Karadayian, Agnese Merlo, Analia Czerniczyniec, Silvia Lores-Arnaiz, Pauline A. Hendriksen, Pantea Kiani, Gillian Bruce, Joris C. Verster

**Affiliations:** 1 Instituto de Bioquímica y Medicina Molecular (IBIMOL), CONICET, Universidad de Buenos Aires, Buenos Aires 1113, Argentina; 2 Division of Pharmacology, Utrecht Institute for Pharmaceutical Sciences, Utrecht University, Universiteitsweg 99, 3584CG Utrecht, The Netherlands; 3 Division of Psychology and Social Work, School of Education and Social Sciences, University of the West of Scotland, Paisley PA1 2BE, UK; 4 Centre for Human Psychopharmacology, Swinburne University, Melbourne, VIC 3122, Australia

**Keywords:** alcohol, hangover, subjective intoxication, COVID-19, smoking, social interactions, students, Argentina

## Abstract

In Argentina, the 2019 coronavirus disease (COVID-19) pandemic led to serious changes to social interaction, health, economy, and education. Argentina experienced two extensive lockdown periods. University education remained virtual for almost two academic years. The purpose of the present work was to analyze the impact of the COVID-19 lockdowns in Argentina on alcohol consumption, hangover severity and smoking among university students in Buenos Aires. A retrospective online survey was conducted in 2021 among students of the University of Buenos Aires. Participants aged 18–35 years old were asked about the average number of alcoholic drinks and number of drinking days per week, binge drinking occasions, drunkenness, next day hangover severity, number of hangovers per month, and smoking behavior. The results showed that the first and second COVID-19 lockdowns were associated with significant reductions in both weekly alcohol consumption, and hangover severity and subjective intoxication on their heaviest drinking occasions. Males consumed significantly more alcohol than females, and older students (25–35 years old) consumed more alcohol than younger students (18–24 years old). In addition, younger students reduced the number of cigarettes smoked per day during the two lockdown periods while older students exhibited significantly more smoking days per week. In conclusion, the present work in Argentinian students revealed a significant reduction in weekly alcohol consumption, and subjective intoxication and hangover severity on their heaviest drinking occasions during the pandemic lockdown periods.

## 1. Introduction

The 2019 coronavirus disease (COVID-19) pandemic had a negative impact on social interaction, health, economy, and education throughout the world [1,2]. In Argentina, on 19 March 2020, a preventive and mandatory social isolation policy (ASPO, for its acronym in Spanish) was established to manage the COVID-19 pandemic. During this period, which lasted until August 2020, only essential places remained open, and people were confined to their homes and only permitted to go out for basic supplies. ASPO was extended repeatedly with jurisdictional variations depending on the epidemiological situation, and had important consequences on routine habits for the adolescent and young adult populations. For example, bars and restaurants were closed and educational institutions transitioned to online education at the start of the ASPO lockdown. After August 2020, lockdown continued until January 2021, but at this point some approvals were introduced for social recreation, while maintaining preventive and mandatory distancing on top of adopting health measures. The summer of 2021 (January–March 2021) comprised a non-lockdown period and people were allowed to travel on vacation. From April to July 2021, most public health regulations that had been implemented during the first lockdown were nationally re-established due to the increase in SARS-CoV-2 infections. Following this period, daily life returned to ‘normal’ but universities remained closed and teaching continued to be conducted online until the end of 2021.

Several studies from different countries have investigated the negative psychological and social effects of lockdowns on both adolescents and adults, including increased levels of anxiety, depression, and loneliness [3,4]. The impact on alcohol consumption has also been reported. However, information on alcohol consumption during the COVID-19 pandemic in Argentina is limited. Alomo and colleagues [5] reported an increase of 20% in alcohol consumption in young Argentinian adults (18–24 years old) during the first lockdown period, while only 10% of individuals older than 55 years increased their alcohol consumption. During ASPO, among adults (35–45 years old), a 45% increase in the consumption of alcoholic beverages was reported in the Buenos Aires Metropolitan Area [6]. The authors attributed this behavior to the increase in free time and the absence of a stable routine. Furthermore, the number of people who drank alcohol every day tripled. Sciannameo et al. [7] observed that of the total of individuals surveyed, 66.3% reported drinking alcohol regularly, and 40.1% increased alcohol beverage consumption during ASPO. In a study conducted by Gomez et al. (2020) it was observed that 70% of the people surveyed consumed alcoholic beverages during quarantine [8]. In addition, an increase in frequency of alcohol consumption (41%) and an increase in the volume of alcohol consumed (33%) was reported by people surveyed [9].

While most surveys related to alcohol consumption during the pandemic investigated the general adult population, two studies focused specifically on Argentinean college students. A significant decrease in alcohol consumption during the first COVID-19 lockdown was reported by Leonangeli et al. [10]. In addition, a decrease in social and enhancement motives for alcohol consumption was shown during the quarantine. In addition, alcohol-related negative events among Argentine university students decreased significatively, probably due to the reduction in social meetings and the closure of drinking places [11]. Another study conducted in Argentina among the general population found an overall small reduction in usual alcohol intake, and a more profound reductions in binge drinking [12]. The greatest reductions in alcohol consumption were reported among the 14 to 24 year old age group. Income was shown to be a factor that influenced alcohol consumption. That is, reductions in alcohol consumption were more profound in those whose income remained equal, or lowered, during the first three months of the COVID-19 pandemic.

A recent nation-wide study in Uruguay revealed that among participants aged 15–65 years, alcohol (52%) and tobacco (33%) were the substances with the highest level of habitual consumption, with women being more likely to increase tobacco use during the COVID-19 pandemic compared with men [13]. Another study reported that among healthcare workers from Latin America, smoking tobacco increased between 2020 and 2021 [14]. In contrast, a study in cardiometabolic patients in Latin America revealed that women reported lower tobacco use during the pandemic [15].

There are no scientific reports about pandemic effects on smoking in Argentina. Related to alcohol consumption, Argentinean studies are limited to assessments of weekly alcohol consumption, and usually only cover the first lockdown period. In addition, no information has been collected on subjective intoxication and the frequency and severity of hangovers during lockdown and non-lockdown periods. Thus, the present work aimed to study the impact of the COVID-19 pandemic in Argentina on alcohol consumption, hangovers, and smoking, among university students in Buenos Aires.

## 2. Methods

In 2021, a retrospective online survey using QuestionPro Survey Software (QuestionPro Inc., Seattle, WA, USA) was completed by university students in Buenos Aires, Argentina, between July and November 2021. At this time, the country had left lockdown and life was returning to normal. Participants were invited via university email to complete the survey, which examined COVID-19 lockdown effects on mood, academic functioning, alcohol consumption, smoking, and perceived immune fitness. They could take part in the study if they were between 18 and 35 years old and students at the University of Buenos Aires. The study was approved by the Ethics Board of the University of the West of Scotland (approval code: 16410). Informed consent was obtained from all participants. A detailed description of the study methodology and the corresponding dataset have been published elsewhere [16]. For the current analysis, only participants that consumed alcohol were included. Demographic data were considered, including age and sex, as well as data on alcohol consumption and smoking.

### 2.1. Alcohol Consumption and Smoking

Participants were asked to report the ‘average number of alcoholic drinks per week’ that they consumed (answer possibilities: 0 to >100) and ‘the number of drinking days per week’ (answer possibilities: 0 to 7 days). Guidance was provided on serving sizes and how to convert these into standard alcoholic drink sizes (units). For liquor and mixed drinks, one shot equaled one unit. One glass of beer (250 mL), and one glass of wine, counted as one unit. One bottle of wine (750 mL) equaled 6 units, and one bottle of liquor (750 mL) equaled 20 units. The items were rated for (1) the period before the COVID-19 pandemic, (2) the first lockdown period (March–December 2020), (3) summer 2021 (January–March 2021, no lockdown), and (4) the second lockdown (April–July 2021). Regarding the heaviest drinking occasions in these time periods, participants reported the number of alcoholic drinks they consumed on such occasions. Participants rated their drunkenness (i.e., subjective intoxication) on a scale ranging from 0 (sober) to 10 (extremely drunk) [17], and next day hangover severity was rated on a scale ranging from 0 (absent) to 10 (extreme) [18]. Participants also reported ‘the number of hangovers per month’ that they experienced (answer possibilities: 0 to 31 days) for the four time periods. For each period, participants reported how many days per week they smoked (answering possibilities: 0 to 7 days), and on average how many cigarettes they smoked per day (answering possibilities: 0 to >100).

### 2.2. Statistical Analysis

Data were analyzed with SPSS (IBM Corp. Released 2013. IBM SPSS Statistics for Windows, Version 28.0. Armonk, NY, USA: IBM Corp.). Mean and standard deviation (SD) were computed for all variables. Within-subject comparisons of the mood assessments of the four time points were conducted with the related samples Friedman’s two-way analysis of variance by rank test. A Bonferroni’s correction was applied, and differences were considered significant if *p* < 0.017. Between-group comparisons (male versus female, and 18–24 years old versus 25–35 years old) were conducted with the independent samples Mann–Whitney U test. Differences between the groups were considered significant if *p* < 0.05.

## 3. Results

A total of N = 508 students participated in the study (N = 149 males and N = 359 females; N = 391 of the young age group and N = 117 of the older age group). Of them, N= 258 (53.5%) consumed alcohol. The sample of alcohol consumers comprised N = 72 males and N = 186 females. Their mean (SD) age was 23.0 (3.5) years old. The young age group (18–24 years old) comprised N = 188 participants and the older age group (25–35 years old) comprised 70 participants. N = 107 (24.7%) of the sample reported smoking tobacco. The sample of smokers consisted of N = 47 males and N = 60 females. Their mean (SD) age was 22.5 (3.8) years old. Among smokers, the young age group (18–24 years old) comprised N = 85 participants and the older age group (25–35 years old) comprised 22 participants.

### 3.1. Alcohol Consumption

**Figure 1:**
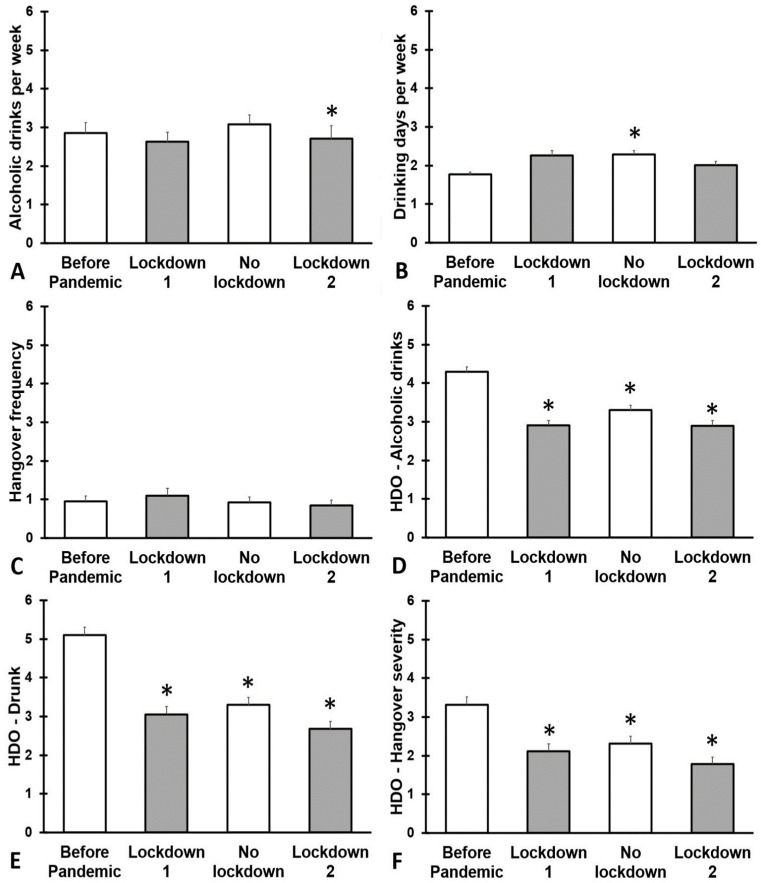
Alcohol consumption and hangovers during the 2019 coronavirus disease (COVID-19) pandemic. Means and standard errors are shown for: (1) the period before the COVID-19 pandemic, (2) the first lockdown period (March–December 2020), (3) summer 2021 (January–March 2021, no lockdown), and (4) the second lockdown (April–July 2021). Data are shown for: (**A**) number of alcoholic drinks consumed per week, (**B**) drinking days per week, (**C**) hangover frequency per month, (**D**) number of alcoholic drinks consumed on the heaviest drinking occasions, (**E**) drunkenness (subjective intoxication) on the heaviest drinking occasions, and (**F**) hangover severity the day after the heaviest drinking occasion. Differences from ‘before the COVID-19 pandemic” are considered significant, after Bonferroni’s correction, if *p* < 0.017, and are indicated by *. Abbreviation: HDO = heaviest drinking occasion.

The outcomes on alcohol consumption are summarized in Figure 1 and Table A1.

Regarding weekly alcohol consumption, the number of alcoholic drinks consumed during the second lockdown was significantly lower compared with before the COVID-19 pandemic (*p* = 0.005). Compared with before the COVID-19 pandemic, significantly more drinking days were reported for the no lockdown period (*p* < 0.001). Looking at the heaviest drinking occasion, compared with before the COVID-19 pandemic, for all assessment periods, participants reported that they consumed significantly less alcohol (*p* < 0.001), were significantly less drunk (*p* < 0.001), and had a significantly less severe next day hangover (*p* < 0.001).

**Figure 2:**
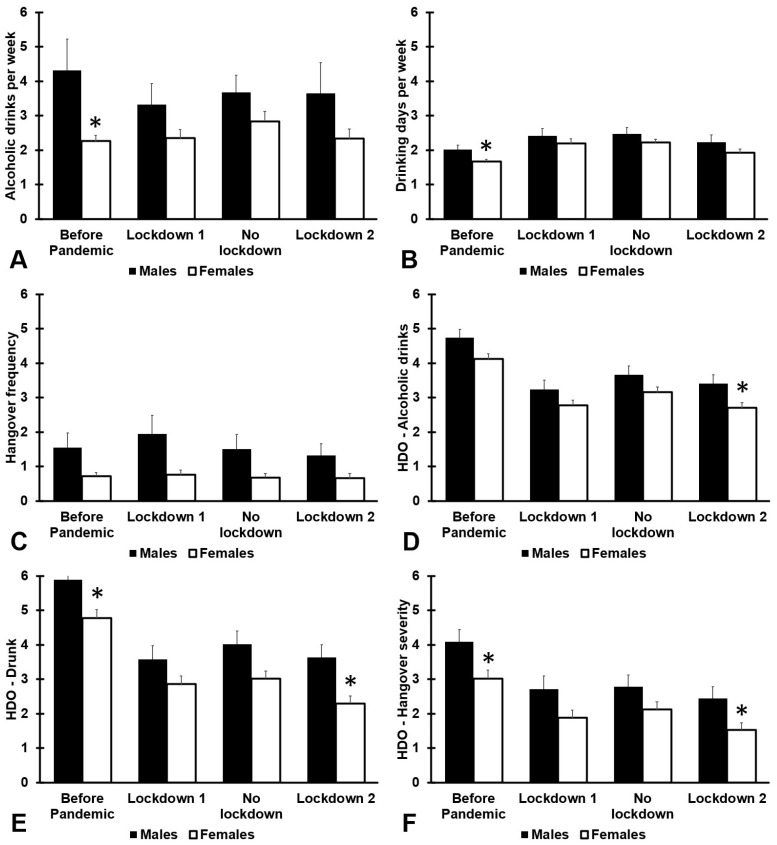
Sex differences in alcohol consumption and hangovers during the COVID-19 pandemic. Means and standard errors are shown for: (1) the period before the COVID-19 pandemic, (2) the first lockdown period (March–December 2020), (3) summer 2021 (January-March 2021, no lockdown), and (4) the second lockdown (April–July 2021). Data are shown for: (**A**) number of alcoholic drinks consumed per week, (**B**) drinking days per week, (**C**) hangover frequency per month, (**D**) number of alcoholic drinks consumed on the heaviest drinking occasions, (**E**) drunkenness (subjective intoxication) on the heaviest drinking occasions, and (**F**) hangover severity the day after the heaviest drinking occasion. Differences between males and females are considered significant, after Bonferroni’s correction, if *p* < 0.0125, and are indicated by *. Abbreviation: HDO = heaviest drinking occasion.

Figure 2 and Table A2 show the results of alcohol consumption according to sex. Before the COVID-19 pandemic, males consumed more alcohol per week than females (*p* = 0.004), reported more drinking days per week (*p* = 0.011), and experienced more pronounced consequences such as drunkenness (*p* = 0.012) and hangover severity (*p* = 0.008). For the second lockdown, participants reported that they consumed significantly fewer alcoholic drinks on their heaviest drinking occasion (*p* = 0.007), and a corresponding significantly lower level of drunkenness (*p* < 0.001) and next day hangover severity (*p* = 0.001).

**Figure 3:**
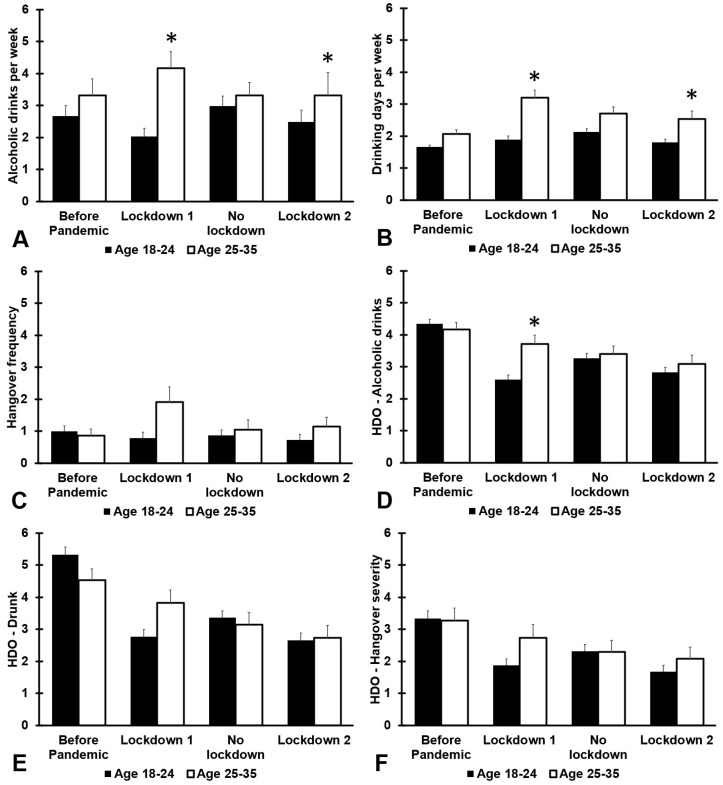
Age differences in alcohol consumption and hangovers during the COVID-19 pandemic. Means and standard errors are shown for: (1) the period before the COVID-19 pandemic, (2) the first lockdown period (March–December 2020), (3) summer 2021 (January–March 2021, no lockdown), and (4) the second lockdown (April–July 2021). Data are shown for: (**A**) number of alcoholic drinks consumed per week, (**B**) drinking days per week, (**C**) hangover frequency per month, (**D**) number of alcoholic drinks consumed on the heaviest drinking occasions, (**E**) drunkenness (subjective intoxication) on the heaviest drinking occasions, and (**F**) hangover severity the day after the heaviest drinking occasion. Differences between the age groups are considered significant, after Bonferroni’s correction, if *p* < 0.0125, and are indicated by *. Abbreviation: HDO = heaviest drinking occasion.

Figure 3 and Table A3 show the results on alcohol consumption according to age group. Before the COVID-19 pandemic, there were no significant differences between the two age groups. Compared with before the COVID-19 pandemic, for the first and second lockdown period, the older age group reported significantly more drinking days per week (*p* < 0.001 and *p* = 0.009, respectively) and number of alcoholic drinks consumed per week (*p* < 0.001 and *p* = 0.008, respectively). With regard to the heaviest drinking occasion, for the first lockdown, older participants reported that they consumed significantly more alcoholic drinks than the younger age group (*p* < 0.001). No other differences between the age groups were statistically significant.

### 3.2. Smoking

**Figure 4:**
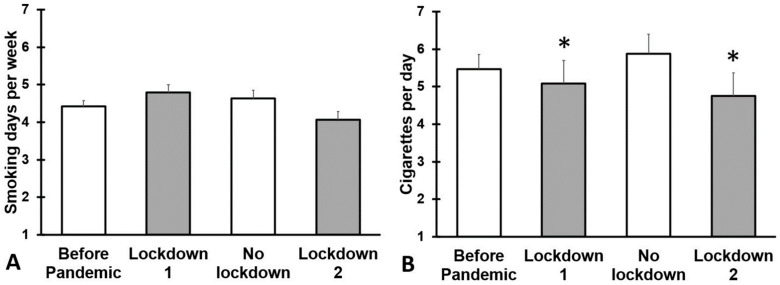
Smoking during the COVID-19 pandemic. Means and standard errors are shown for: (1) the period before the COVID-19 pandemic, (2) the first lockdown period (March–December 2020), (3) summer 2021 (January–March 2021, no lockdown), and (4) the second lockdown (April–July 2021). Data are shown for: (**A**) number of smoking days per week, and (**B**) number of cigarettes smoked per day. Differences from ‘before the COVID-19 pandemic’ are considered significant, after Bonferroni’s correction, if *p* < 0.017, and are indicated by *.

The sample of smokers comprised N = 107 participants. Their outcomes on smoking are summarized in Figure 4 and Table A4. No significant differences were found for the number of smoking days before and during the COVID-19 pandemic. However, for the first and second lockdown period, participants reported smoking significantly fewer cigarettes per day (*p* = 0.003 and *p* < 0.001, respectively).

**Figure 5:**
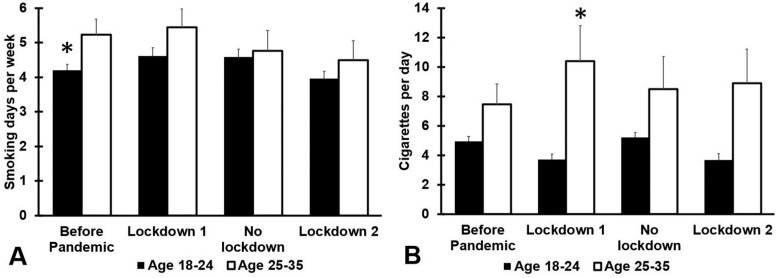
Smoking during the COVID-19 pandemic, according to age group. Means and standard errors are shown for: (1) the period before the COVID-19 pandemic, (2) the first lockdown period (March–December 2020), (3) summer 2021 (January–March 2021, no lockdown), and (4) the second lockdown (April–July 2021). Data are shown for: (**A**) number of smoking days per week, and (**B**) number of cigarettes smoked per day. Differences between 18–24 year old and 25–35 year old participants are considered significant, after Bonferroni’s correction, if *p* < 0.0125, and are indicated by *.

No significant sex differences were found with regard to smoking (see Table A5). Smoking outcomes according to age are summarized in Figure 5 and Table A6.

Before the COVID-19 pandemic, the 25–35 year old age group reported significantly more smoking days per week (*p* = 0.011) compared with the younger age group. This difference was not significant during the pandemic. Overall, older participants smoked more cigarettes per day than younger participants. However, the difference between the age groups was statistically significant only for the first lockdown period (*p* = 0.001).

Of interest was the age difference in smoking behavior before and during the COVID-19 pandemic. The young age group showed a significant reduction in the number of cigarettes smoked per day during the two lockdown periods (*p* < 0.001) In contrast, the 25–35 years old group reported no significant changes in smoking behavior.

## 4. Discussion

In Argentina, the first and second COVID-19 lockdowns were associated with significant reductions in both weekly alcohol consumption, hangover severity and subjective intoxication on their heaviest drinking occasions. Males consumed significantly more alcohol than females, and older students (25–35 years old) consumed more alcohol than younger students (18–24 years old). In addition, younger students reduced the number of cigarettes smoked per day during the two lockdown periods while older students exhibited significantly more smoking days per week.

The reduction in alcohol consumption and smoking could be linked to the pandemic and its social isolation, which negatively affected the labor situation and income level among many people. In addition, the lockdown measures limited access to alcohol as many drinking venues were closed. More drinking days were reported for the no lockdown period compared with before the COVID-19 pandemic. This could be a rebound effect due to the long duration of the first lockdown in Argentina. It was recently proposed that changes in alcohol consumption patterns might have followed one of two possible paths: either people reduced their consumption due to the lack of economic resources, or increased it due to an additional source of psychological distress [19]. Regarding heaviest drinking occasions, less alcohol was consumed in all the pandemic stages and this was accompanied by less drunkenness and hangover severity. Our data do not support the findings by Garcia-Cerde et al. [20], who reported that quarantine was correlated with higher frequency of heavy episodic drinking in Latin America.

It was expected that changes in consumption during the COVID-19 pandemic may differ among population groups. Indeed, differences according to sex were found between lockdown periods. Before the COVID-19 pandemic, males consumed more alcohol per week than females, reported more drinking days per week and experienced more pronounced consequences such as drunkenness and hangover severity. In addition, age differences were found across pandemic stages. For the first and second lockdown periods, the older students reported significantly more drinking days per week and number of alcoholic drinks consumed per week, compared with younger students. Regarding the heaviest drinking occasion, for the first lockdown, older students reported consuming significantly more alcoholic drinks than the younger age group. It was expected that younger adults’ drinking would decrease, since social meetings and other events associated with youth drinking were restricted or forbidden during lockdowns. For example, a study from Poland showed that people who decreased their alcohol consumption during the pandemic were more likely to be of younger age [21]. Similarly, a study performed with Canadian adolescents showed a significant decrease in binge drinking during the pandemic [22]. Additionally, the 18–24 year old group showed a significant reduction in the number of cigarettes smoked per day during the two lockdown periods. This finding was in line with our study’s observed reductions in alcohol consumption. While there were no sex differences in smoking behavior, older students reported significantly more smoking days per week than the younger age group.

It is important to recall that the limitations of the study include the retrospective nature of the survey and associated risk of recall bias. In addition, there might have been a self-selection bias concerning students that did and did not participate in the study, that may also in some ways explain the higher proportion of females The current study comprised a convenience sample of students. The sex distribution was, therefore, not balanced. A technical error in the survey design did not allow students to select more than seven drinks on their heaviest drinking occasion. Although the reported weekly alcohol consumption in this study was low, it might be possible that some students could have consumed more than seven drinks and were not able to indicate this. Finally, we conducted a separate analysis to evaluate sex and age effects. Alternatively, we could have conducted a full analysis that controlled for these variables. However, we felt it was important to show the direct comparisons between males and females (Figure 2) and the age groups (Figure 3 and Figure 5). Notwithstanding these limitations, the present study is of importance for Argentinian data since the results provide, for the first time, a deeper insight into alcohol consumption, hangover severity and smoking in students during the COVID-19 pandemic. Ongoing additional reports about analyses of mood, immune fitness and academic performance during the pandemic in the same university student population will be analyzed in the context of the data presented in this study.

## 5. Conclusions

COVID-19 lockdowns in Argentina were associated with significant reductions in both weekly alcohol consumption and smoking, as well as hangover severity and subjective intoxication on the heaviest drinking occasions. Sex and age differences were found, showing that males consumed more alcohol than females, and older students exhibited a significantly higher alcohol intake than younger students. Lockdown periods were also associated with significantly reduced smoking behavior.

## Data Availability

The data are published as open access in the journal MDPI Data and are available online as supplement to reference [16].

## Appendix A

**Table A1:** Alcohol consumption.

Overall Assessments	Mean (SD)					Pairwise Comparisons		
Time Period	B	L1	NL	L2	Overall	B vs. L1	B vs. NL	B vs. L2
Number of alcoholic drinks per week	2.9 (4.3)	2.6 (3.7)	3.1 (3.8)	2.7 (5.1)	<0.001 *	0.022	0.090	0.005 *
Drinking days per week	1.8 (0.9)	2.3 (1.8)	2.3 (1.4)	2.0 (1.5)	<0.001 *	0.063	<0.001 *	0.540
Hangover frequency per month	1.0 (2.2)	1.1 (2.8)	0.9 (2.2)	0.9 (2.1)	0.017 *	0.195	0.188	0.038
HDO—number of alcoholic drinks	4.3 (1.9)	2.9 (2.0)	3.3 (1.9)	2.9 (2.0)	<0.001 *	<0.001 *	<0.001 *	<0.001 *
HDO—subjective intoxication	5.1 (3.0)	3.1 (3.0)	3.3 (2.9)	2.7 (2.9)	<0.001 *	<0.001 *	<0.001 *	<0.001 *
HDO—hangover severity	3.3 (3.1)	2.1 (2.9)	2.3 (2.8)	1.8 (2.7)	<0.001 *	<0.001 *	<0.001 *	<0.001 *

Mean, standard deviation (SD, between brackets), and *p*-values are shown. Significant differences between before the COVID-19 pandemic (B) and the other time periods are indicated by *. Pairwise comparisons were computed if the main effect was significant (*p* < 0.05), and considered significant if *p* < 0.017, after Bonferroni’s correction for multiple comparisons. Abbreviations: B = before the COVID-19 pandemic, L1 = lockdown 1, NL = no lockdown, L2 = lockdown 2, HDO = heaviest drinking occasion, COVID-19 = coronavirus disease 2019.

**Table A2:** Alcohol consumption according to sex.

Time Period	Before COVID-19		Lockdown 1		No Lockdown		Lockdown 2	
Sex	Males	Females	Males	Females	Males	Females	Males	Females
Number of alcoholic drinks per week	4.3 (7.0)	2.3 (2.2) ^†^	3.3 (4.7)	2.4 (3.2)	3.7 (4.3)	2.8 (3.5)	3.7 (7.5) *	2.3 (3.7)
Drinking days per week	2.0 (1.0)	1.7 (0.8) ^†^	2.4 (1.8)	2.2 (1.8)	2.5 (1.6)	2.2 (1.3) *	2.2 (1.8)	1.9 (1.4)
Hangover frequency per month	1.6 (3.5)	0.7 (1.2)	1.9 (4.3)	0.8 (1.8)	1.5 (3.5)	0.7 (1.3)	1.3 (2.7)	0.7 (1.7)
HDO—number of alcoholic drinks	4.7 (1.9)	4.1 (1.9)	3.2 (2.2) *	2.8 (1.9) *	3.7 (2.0) *	3.2 (1.9) *	3.4 (2.0) *	2.7 (1.9) ^†,^*
HDO—subjective intoxication	5.9 (2.8)	4.8 (3.0) ^†^	3.6 (3.2) *	2.9 (3.0) *	4.0 (3.1) *	3.0 (2.8) *	3.6 (3.0) *	2.3 (2.7) ^†,^*
HDO—hangover severity	4.1 (2.9)	3.0 (3.1) ^†^	2.7 (3.1) *	1.9 (2.8) *	2.8 (2.8) *	2.1 (2.9) *	2.4 (2.8) *	1.5 (2.6) ^†,^*

Mean, standard deviation (SD, between brackets), and *p*-values are shown. Significant differences between males and females (*p* < 0.0125, after Bonferroni’s correction) are indicated by ^†^. Significant differences between before the COVID-19 pandemic (B) and the other time periods are indicated by *. Pairwise comparisons were computed if the main effect was significant (*p* < 0.05), and considered significant if *p* < 0.017, after Bonferroni’s correction for multiple comparisons. Abbreviations: HDO = heaviest drinking occasion, COVID-19 = coronavirus disease 2019.

**Table A3:** Alcohol consumption according to age group.

Time Period	Before COVID-19		Lockdown 1		No Lockdown		Lockdown 2	
Age Group (Years)	18–24	25–35	18–24	25–35	18–24	25–35	18–24	25–35
Number of alcoholic drinks per week	2.7 (4.3)	3.3 (4.2)	2.0 (3.4) *	4.2 (4.1) ^†^	3.0 (4.0)	3.3 (3.2)	2.5 (4.8) *	3.3 (5.9) ^†^
Drinking days per week	1.7 (0.8)	2.1 (1.1)	1.9 (1.6)	3.2 (2.0) ^†,^*	2.1 (1.3) *	2.7 (1.6)	1.8 (1.3)	2.5 (1.9) ^†,^*
Hangover frequency per month	1.0 (2.3)	0.9 (1.7)	0.8 (2.3) *	1.9 (3.7)	0.9 (2.1)	1.1 (2.4)	0.7 (2.0) *	1.2 (2.3)
HDO—number of alcoholic drinks	4.3 (2.0)	4.2 (1.8)	2.6 (1.8) *	3.7 (2.3) ^†^	3.3 (1.9) *	3.4 (2.0) *	2.8 (1.9) *	3.1 (2.1) *
HDO—subjective intoxication	5.3 (3.1)	4.5 (2.8)	2.8 (2.9) *	3.8 (3.2)	3.4 (2.9) *	3.2 (3.0) *	2.7 (2.8) *	2.7 (3.0) *
HDO—hangover severity	3.3 (3.1)	3.3 (3.1)	1.9 (2.8) *	2.7 (3.3)	2.3 (2.8) *	2.3 (3.0)	1.7 (2.5) *	2.1 (3.0)

Mean, standard deviation (SD, between brackets), and *p*-values are shown. Significant differences between 18–24 and 25–35 year old groups (*p* < 0.0125, after Bonferroni’s correction) are indicated by ^†^. Significant differences between before the COVID-19 pandemic (B) and the other time periods are indicated by *. Pairwise comparisons were computed if the main effect was significant (*p* < 0.05), and considered significant if *p* < 0.017, after Bonferroni’s correction for multiple comparisons. Abbreviations: HDO = heaviest drinking occasion, COVID-19 = coronavirus disease 2019.

## Appendix B

**Table A4:** Smoking.

Overall Assessments	Mean (SD)					Pairwise Comparisons		
Time Period	B	L1	NL	L2	Overall	B vs. L1	B vs. NL	B vs. L2
Number of cigarettes smoked per day	5.5 (4.0)	5.1 (6.3)	5.9 (5.4)	4.8 (6.4)	<0.001 *	0.003 *	0.213	<0.001 *
Smoking days per week	4.4 (1.7)	4.8 (2.2)	4.6 (2.2)	4.1 (2.2)	0.005 *	0.095	0.412	0.223

Mean, standard deviation (SD, between brackets), and *p*-values are shown. Significant differences between before the COVID-19 pandemic (B) and the other time periods are indicated by *. Pairwise comparisons were computed if the main effect was significant (*p* < 0.05), and considered significant if *p* < 0.017, after Bonferroni’s correction for multiple comparisons. Abbreviations: B = before the COVID-19 pandemic, L1 = lockdown 1, NL = no lockdown, L2 = lockdown 2, COVID-19 = coronavirus disease 2019.

**Table A5:** Smoking according to sex.

Time Period	Before COVID-19		Lockdown 1		No Lockdown		Lockdown 2	
Sex	Males	Females	Males	Females	Males	Females	Males	Females
Number of cigarettes smoked per day	6.0 (4.3)	5.1 (3.8)	5.3 (5.6)	5.0 (6.9)	6.3 (5.5)	5.5 (5.4)	5.0 (7.5) *	4.5 (5.5)
Smoking days per week	4.4 (1.7)	4.4 (1.7)	4.5 (2.2)	5.0 (2.2)	4.7 (2.3)	4.6 (2.2)	3.7 (2.3) *	4.4 (2.0)

Mean, standard deviation (SD, between brackets), and *p*-values are shown. No significant differences between males and female (*p* < 0.0125, after Bonferroni’s correction) were found. Significant differences between before the COVID-19 pandemic (B) and the other time periods are indicated by *. Pairwise comparisons were computed if the main effect was significant (*p* < 0.05), and considered significant if *p* < 0.017, after Bonferroni’s correction for multiple comparisons. Abbreviations: COVID-19 = coronavirus disease 2019.

**Table A6:** Smoking according to age group.

Time Period	Before COVID-19		Lockdown 1		No Lockdown		Lockdown 2	
Age Group (Years)	18–24	25–35	18–24	25–35	18–24	25–35	18–24	25–35
Number of cigarettes smoked per day	5.0 (3.0)	7.5 (6.4)	3.7 (3.2) *	10.4 (11.1) ^†^	5.2 (3.0)	8.5 (10.1)	3.7 (4.1) *	8.9 (11.0)
Smoking days per week	4.2 (1.5)	5.2 (2.1) ^†^	4.6 (2.1)	5.5 (2.5)	4.6 (2.1)	4.8 (2.8)	4.0 (2.1)	4.5 (2.6)

Mean, standard deviation (SD, between brackets), and *p*-values are shown. Significant differences between 18–24 and 25–35 year old groups (*p* < 0.0125, after Bonferroni’s correction) are indicated by ^†^. Significant differences between before the COVID-19 pandemic (B) and the other time periods are indicated by *. Pairwise comparisons were computed if the main effect was significant (*p* < 0.05), and considered significant if *p* < 0.017, after Bonferroni’s correction for multiple comparisons. Abbreviations: COVID-19 = coronavirus disease 2019.
